# Supplementary material for: O-Mannose Glycosylations Influence E-Cadherin Functional Interactions
Source: Mol Cell Proteomics. 2026 Mar 25;25(5):101559. doi: 10.1016/j.mcpro.2026.101559 (PMC13123603; doi:10.1016/j.mcpro.2026.101559)
Supplement: Supplementary Figures [file mmc1.pdf]

O-mannose glycosylations influence E-cadherin functional interactions

Shaoshuai Xie[1,2], Katarina Madunić[3], Omar G. Rosas Bringas[1,4], Weihua Tian[3,5], Sergey Y. Vakhrushev[3], Hjalmar Permentier[6], Peter Horvatovich[6,†], Adnan Halim[3,†,#], John LaCava[4,†,#]

[1]European Research Institute for the Biology of Ageing, University Medical Center Groningen, 9713 AV Groningen, NL; [2]National Glycoengineering Research Center, Shandong University, 266237 Qingdao, CN; [3]Department of Cellular and Molecular Medicine, Faculty of Health Sciences, Copenhagen Center for Glycomics, University of Copenhagen, DK-2200 Copenhagen, DK; [4]Laboratory of Cellular and Structural Biology, The Rockefeller University, NY 10065, US; [5]Department of Biotechnology and Biomedicine, Section for Medical Biotechnology, Technical University of Denmark, DK-2800 Kgs. Lyngby, DK; [6]Department of Analytical Biochemistry, Groningen Research Institute of Pharmacy, University of Groningen, 9713 GZ Groningen, NL.

[†]Senior author

[#]Correspondence to A.H. (halim@sund.ku.dk) and J.L. (jlacava@rockefeller.edu)

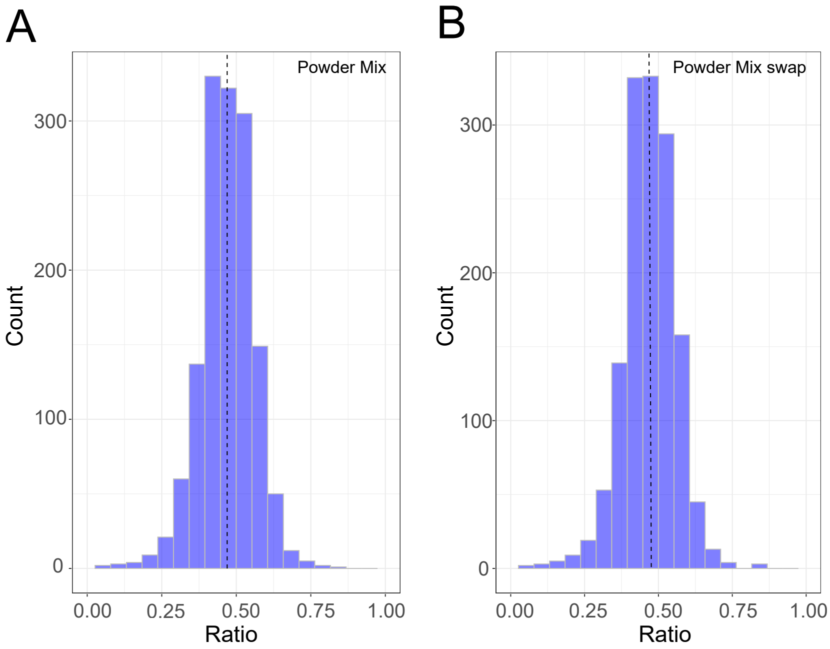

Supplemental Figure 1. Ratio Distribution of Cell Powder Mix. (**A**) Ratio distribution for cell powder mix (light-labeled BG1^{WT} and heavy-labeled BG1$^{CDH1::HA}$). The median ratio for all proteins across 6 replicates is 0.45. The standard deviation (SD) of the median ratio among six replicates is 0.0017. (**B**) Ratio distribution for swapped cell powder mix (heavy-labeled BG1^{WT} and light-labeled BG1$^{CDH1::HA}$). The median ratio for all proteins across 6 replicates is 0.48. The standard deviation (SD) of the median ratio among six replicates is 0.0012.

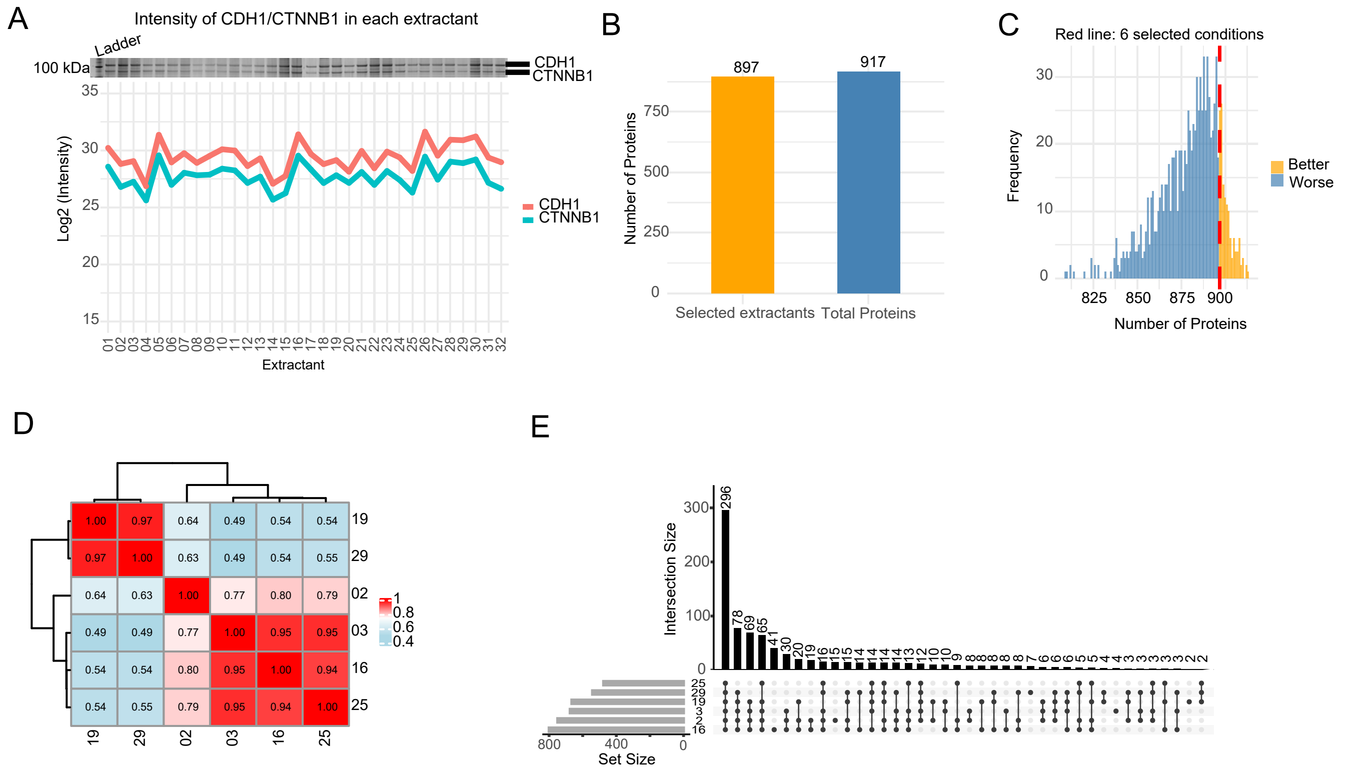

Supplemental Figure 2. Extractant Selection for IP Screening. (**A**) CDH1 and its interactor CTNNB1 intensities in the SDS-PAGE gel (silver stain) and label-free mass spectrometry. (**B)** Number of proteins identified for selected 6 extractants and all 32 extractants. A total of 917 proteins were identified in 32 extractants, while 897 proteins were identified in the 6 selected extractants. (**C**) Distribution of identified protein numbers from 1000 random selections of 6 extractants. The number of proteins for the 6 selected extractants in Fig. 1C is indicated by a red dotted line. (**D**) Correlation of LFQ MS results for 6 selected extractants**.** (**E**) UpSet analysis of proteins identified in the 6 selected extractants. All ms-based analyses use protein intensity values output from Proteome Discoverer ("Abundance"), as per Fig. 1C.

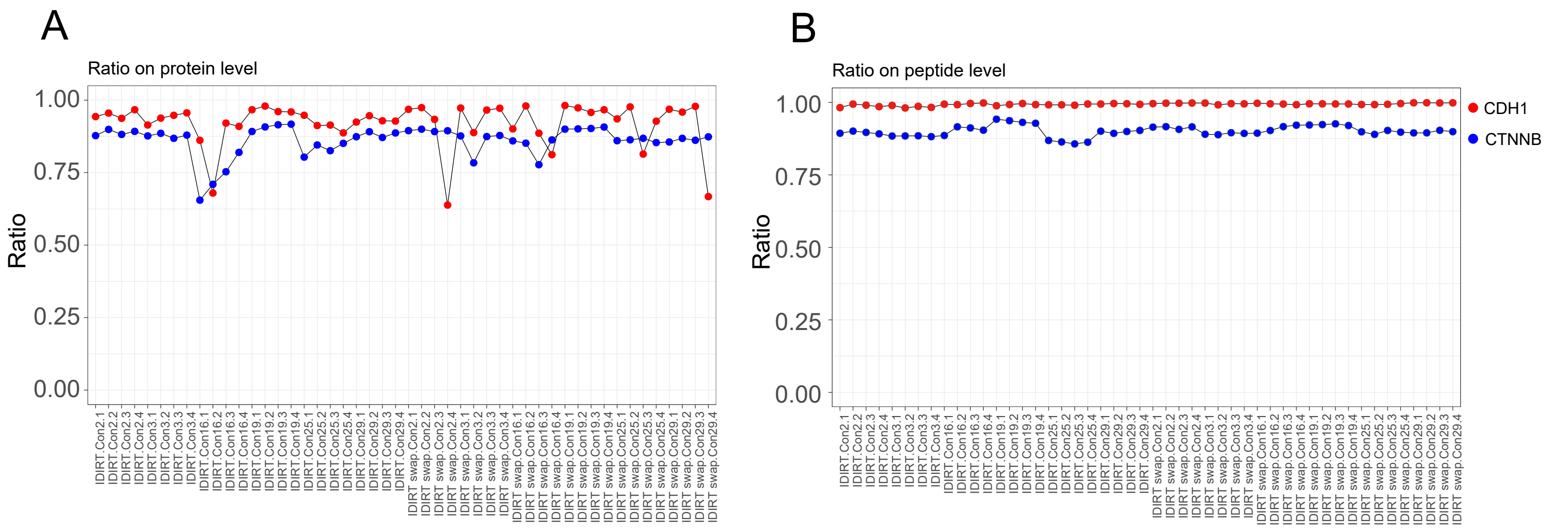

Supplemental Figure 3. I-DIRT Ratios for CDH1 and CTNNB1. (**A**) I-DIRT ratios for CDH1 and CTNNB1 based on protein intensity, quantified using Proteome Discoverer. (**B**) I-DIRT ratios for CDH1 and CTNNB1 based on peptide intensities, quantified using Proteome Discoverer.

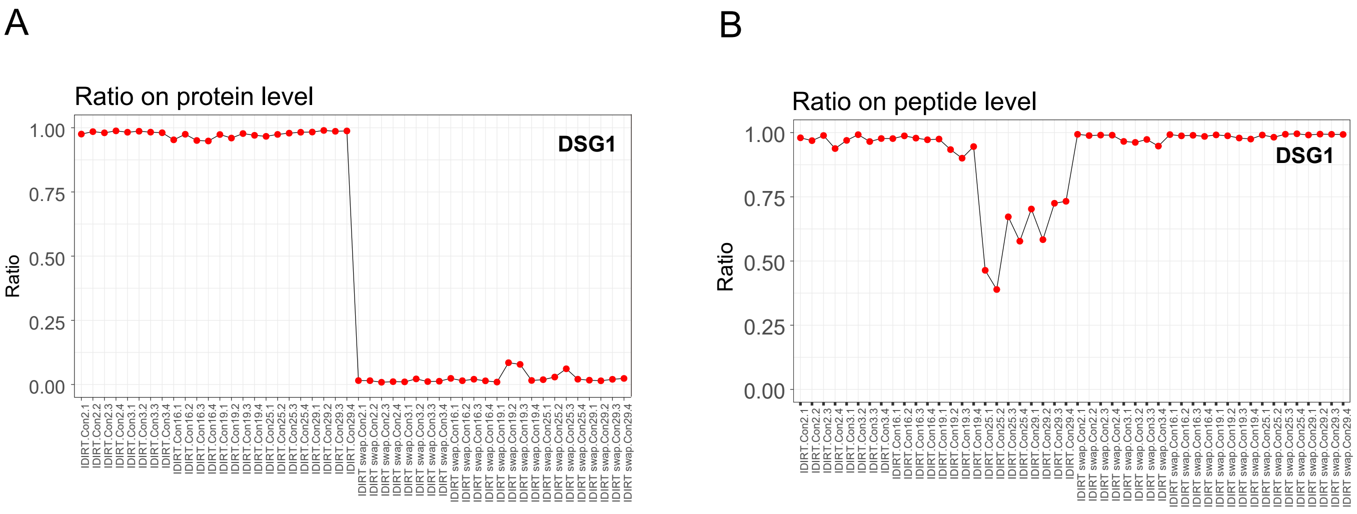

Supplemental Figure 4. I-DIRT ratio of DSG1. (A) I-DIRT ratios for DSG1 based on protein abundance quantified by Proteome Discoverer. (B) I-DIRT ratios for DSG1 based on peptide abundance quantified by Proteome Discoverer.

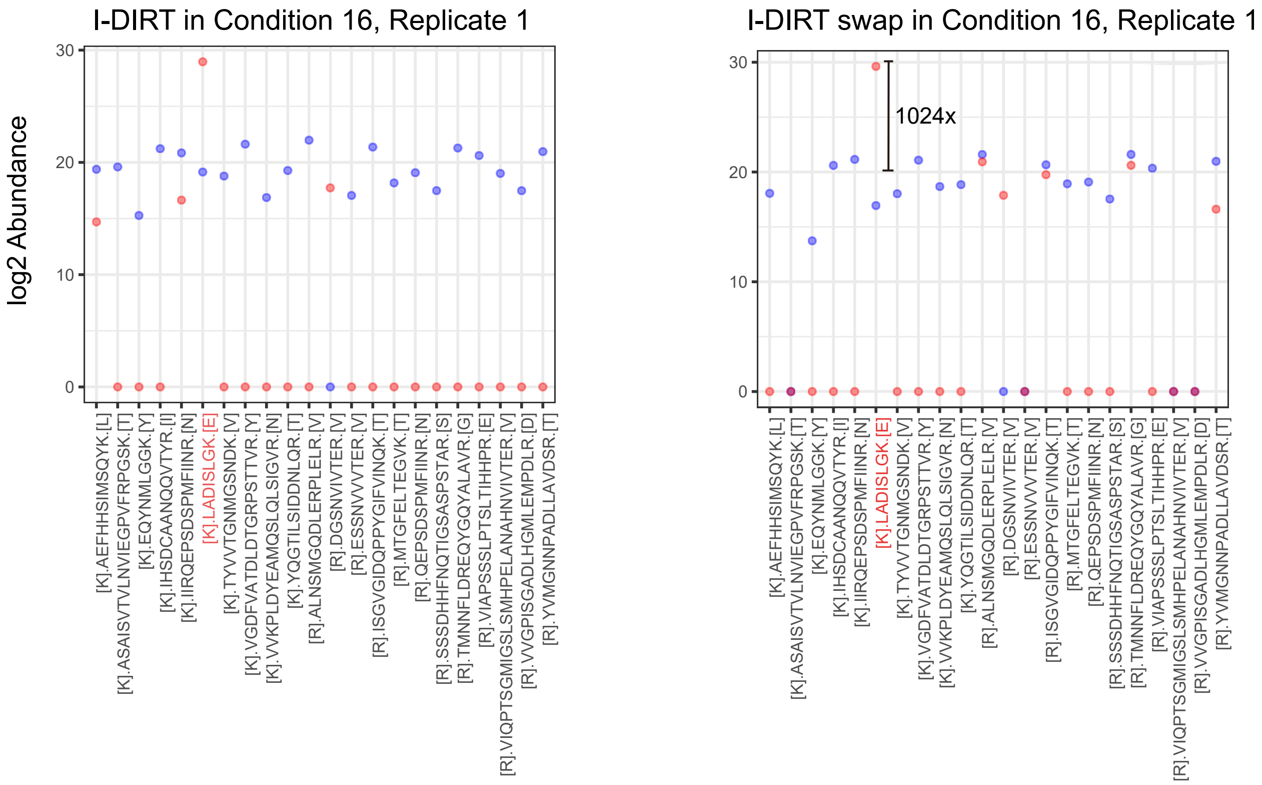

Supplemental Figure 5. Peptide abundance of DSG1 in I-DIRT experiments. One replicate each for condition 16 in I-DIRT and I-DIRT swap experiments is shown respectively. The abundance of a single heavy-labeled peptide (red) is nearly 1000 times higher than that of other peptides, disproportionately contributing to the total protein abundance.

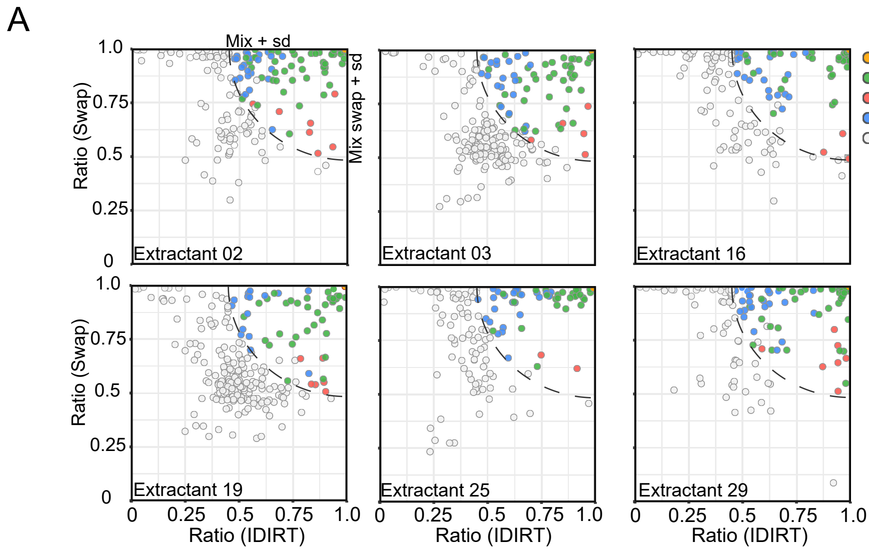

NO.	Buffer	pH	Detergent	Concentratior	Salt	Calcium
2	20mMHEPES	7.4	1%(v/v) TritX100	500mM	KAcet	1mM
3	20mMHEPES	7.4	1%(v/v) TritX100	250mM	KAcet	No
16	40mMTris-HCl	8	20mM CHAPS	500mM	NaCl	No
19	20mMHEPES	7.4	1%(w/v) DDM	250mM	KAcet	No
25	20mMHEPES	7.4	1%(w/v) C12E8	250mM	NaCl	No
29	40mMTris-HCl	8	1%(w/v) C12E8	250mM	NaCl	1mM

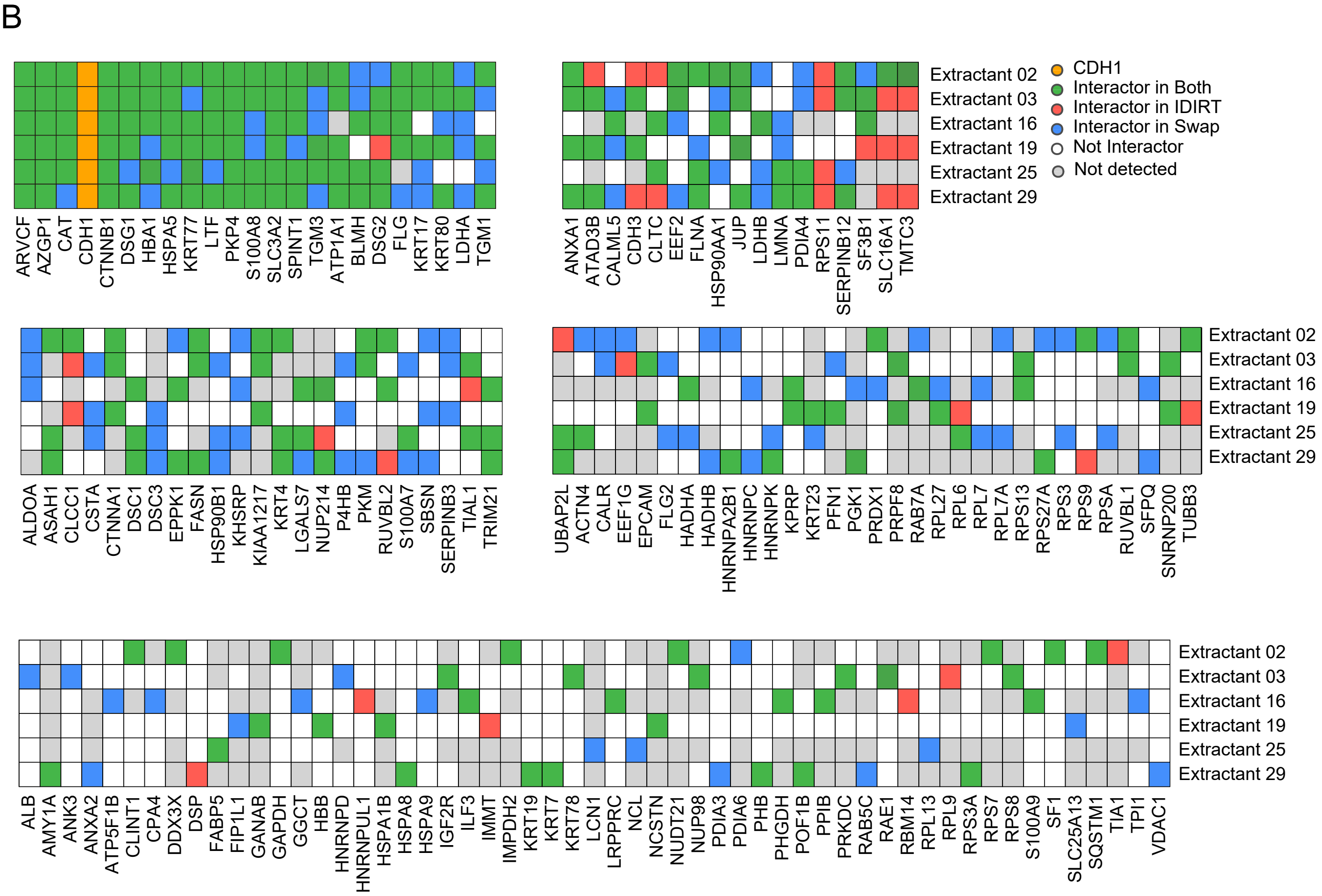

Supplemental Figure 6. I-DIRT specific CDH1 interactors identified in different IP conditions. (**A**) Specific interactors were identified by comparing protein enrichment ratios from the I-DIRT and I-DIRT swap experiments. For each experiment, a Bayesian test was applied to compare the enrichment ratio of a given protein to the median ratio of all proteins in the corresponding bulk proteome (mix or mix swap). Proteins were classified as specific interactors if they met two criteria: a Bayesian Factor (BF) > 3 and location within the significance ellipse. The ellipse's semi-major and semi-minor axes were defined by the median ratio plus or minus one standard deviation (SD) of the bulk proteome, respectively. The table on the right shows the formulation of the selected six extractants. (**B**) Summary of the CDH1 interactome across six IP extractants.

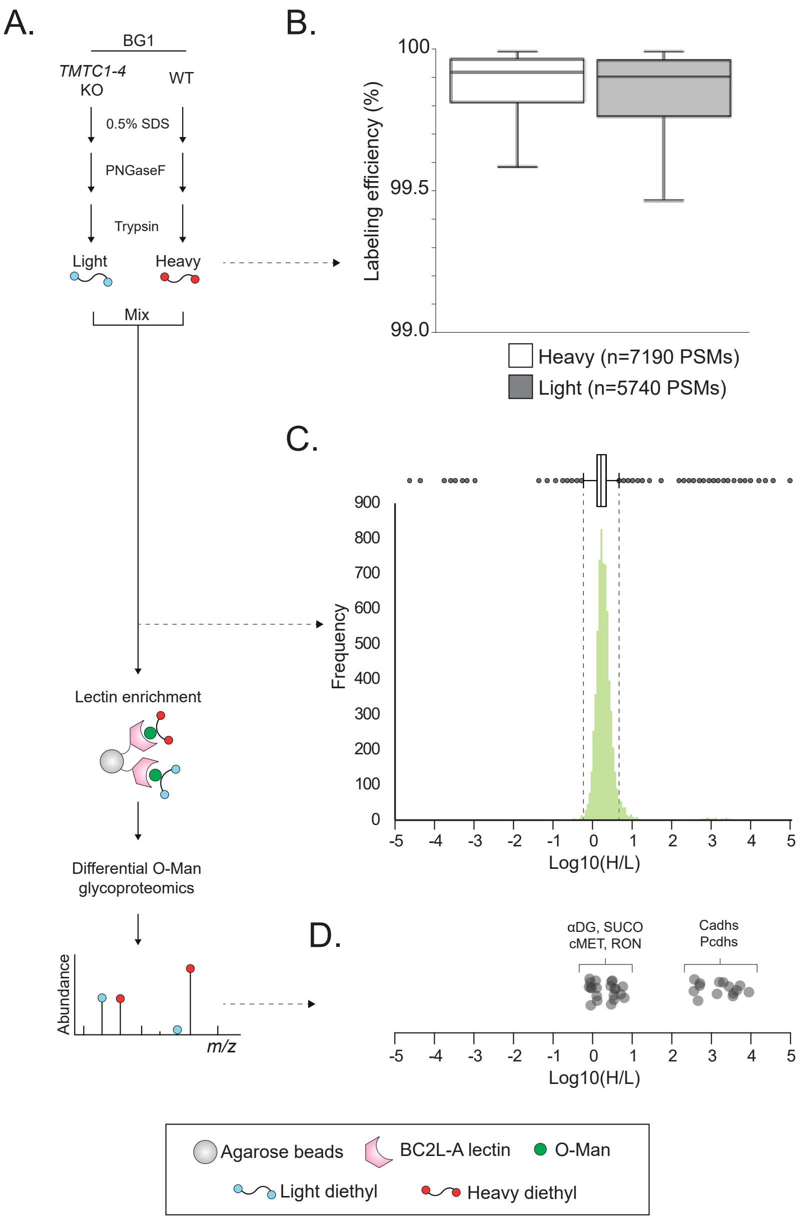

Supplemental Figure 7. TMTC1-4 dependent O-Man glycosylation of cadherins and protocadherins in BG1 cells. (**A**) Workflow for comparative analysis of O-Man glycosylation in BG1^{WT} and BG1$^{KO:\ TMTC1\text{-}4}$ cells as previously described (55). (**B**) Diethyl stable isotope (DEL) labeled total cell tryptic digests were individually analyzed and MS1 peaks of peptide spectral matches (PSMs) were used to assess labeling efficiency (E_L), calculated by E_L = 1- (1/((labeled/unlabeled) +1))). (**C**) Heavy (H) and light (L) labeled tryptic digest were mixed (volume 1:1) and analyzed to assess mixing ratio and proteome variability. The bar chart shows $\log_{10}$ transformed H/L frequencies of identified peptides (n=6664 PSMs). Outliers were determined by Q1-1.5xIQR and Q3+1.5xIQR boundaries (dashed lines). (**D**) BC2L-A lectin enriched fractions were analyzed as previously described (55), and relative abundances of identified *O*-Man glycopeptides plotted as $\log_{10}$(H/L) ratios. The dot-plot shows a group of glycopeptides (<10-fold change) originating from αDG and SUCO (POMT1/POMT2 substrates) as well as cMET and RON receptors (TMEM260 substrates), while the canonical TMTC1-4 substrates including cadherins (Cadhs) and protocadherins (Pcdhs) show >100-fold change, thus demonstrating that *TMTC1-4* KO abolishes cadherin-specific *O*-Man glycosylation in BG1 cells.

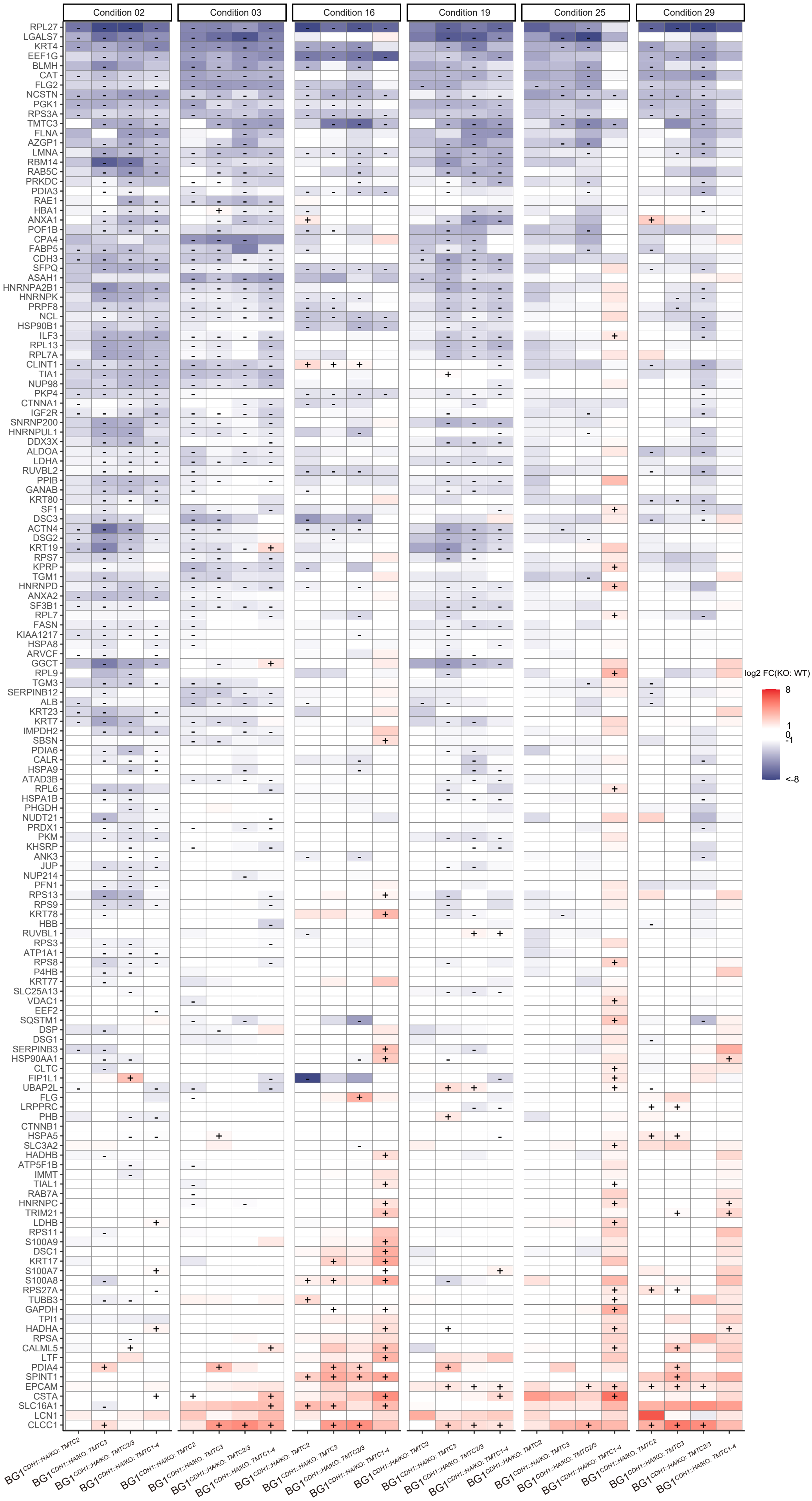

Supplemental Figure 8. The *O*-Man dependent E-cadherin interactome. The heatmap displays the log_2FC of CDH1 interactors across four *TMTC* KO cell lines under six IP conditions. Rows represent interactors, while columns represent IP

conditions for each KO cell line. Red indicates up-regulated interactors, and blue indicates down-regulated interactors, with color intensity reflecting the magnitude of change. Symbols indicate statistical significance, with "+/-" representing $\log_2$FC >1 or < -1 and $p < 0.05$.

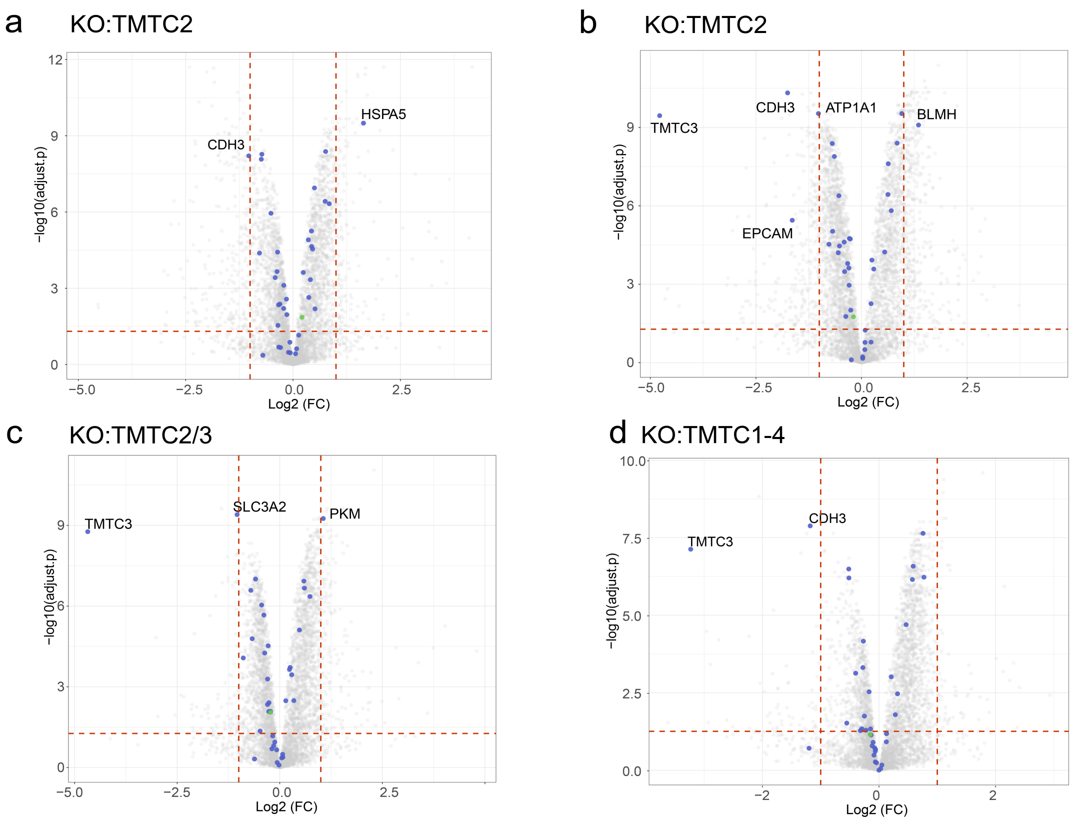

Supplemental Figure 9. Whole cell lysate proteome analysis for different cell lines. The I-DIRT specific CDH1 interactors are colored blue. CDH1 is colored green. Only proteins with abundance changes in the given cell extracts are labeled with the Uniprot gene symbol.

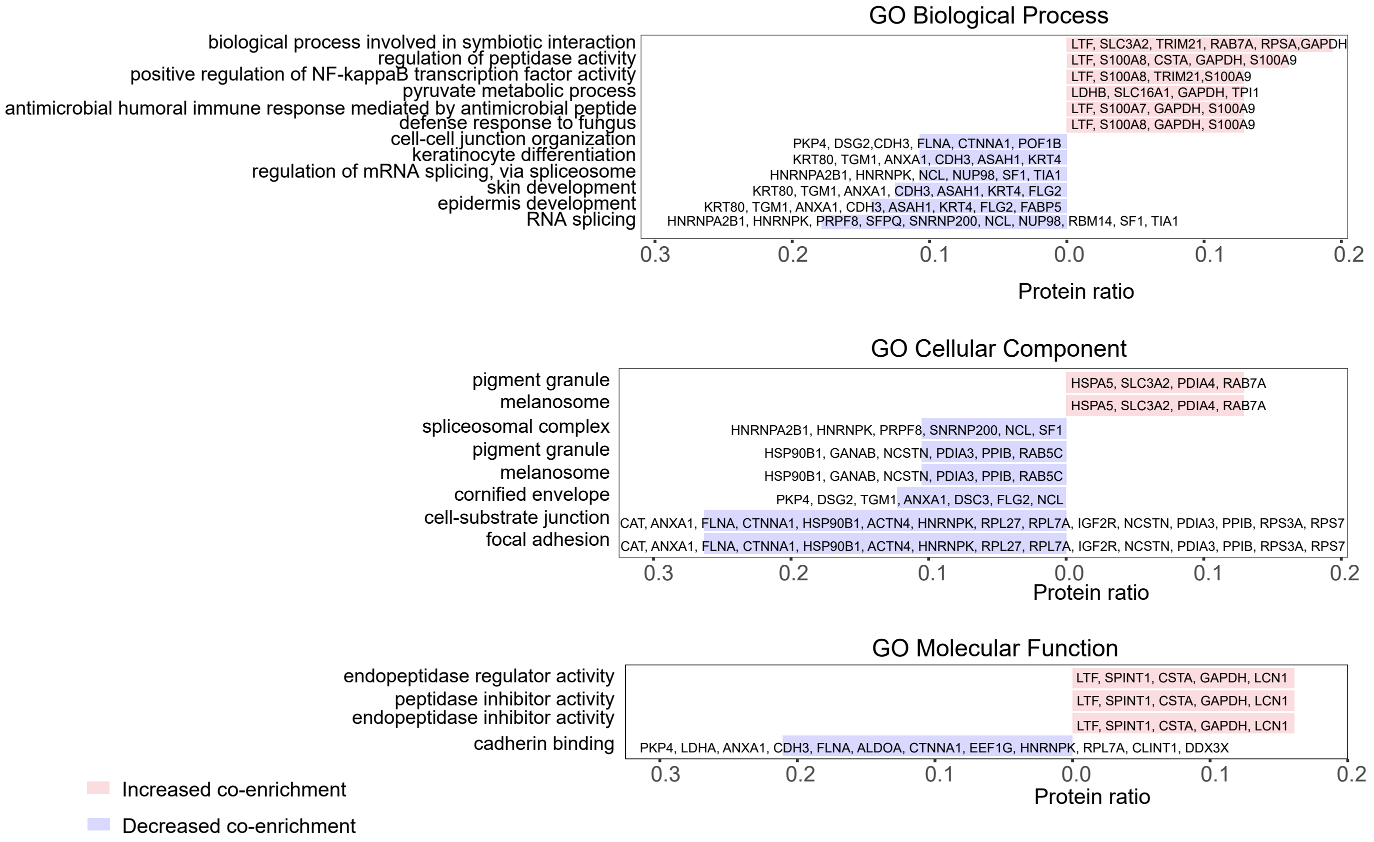

Supplemental Figure 10. Gene Ontology (GO) analysis was performed on CDH1 interactors identified as O-Man-dependent. The results show enriched terms for Biological Process (BP), Cellular Component (CC), and Molecular Function (MF), highlighting the key roles of these interactors in adhesion, signaling, and protein stability. Categories are presented with a protein ratio greater than 10% and a p-value < 0.01.

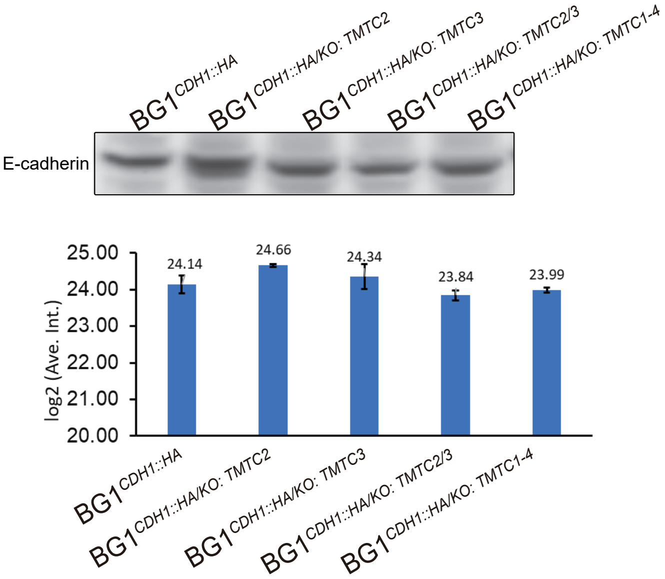

Supplemental Figure 11. Expression of E-cadherin in different cell lines. The expression of E-cadherin was quantified via Western blot analysis (n = 3). Equally 20 µg of lystates were loaded in each lane.

20 µg of cell lysate (processed following Bulk protein extraction from cells, see Methods) was diluted in 1× LDS (Invitrogen), reduced with dithiothreitol (10min, 90◦C), and resolved on NuPAGE Bis-Tris 4–12% gels (Invitrogen) using 1× MOPS buffer (Invitrogen). Proteins were transferred (1.5h, 90 V) to a methanol-activated PVDF membrane with Tris-Glycine transfer buffer (25 mM Tris, 192 mM glycine, 10% ethanol). The membrane was blocked for 1 h at room temperature (RT) with gentle agitation in TBST containing 5% (w/v) non-fat milk, followed by incubation with an HA-Tag Monoclonal Antibody (#26183, Thermo Fisher, 0.2 µg/mL in TBST containing 5% (w/v) BSA and 0.02% (w/v) sodium azide). After washing with TBST, HA-tagged CDH1 was detected using an HRP-conjugated anti-mouse secondary antibody (Thermo Fisher, 1:10,000 dilution in TBST).

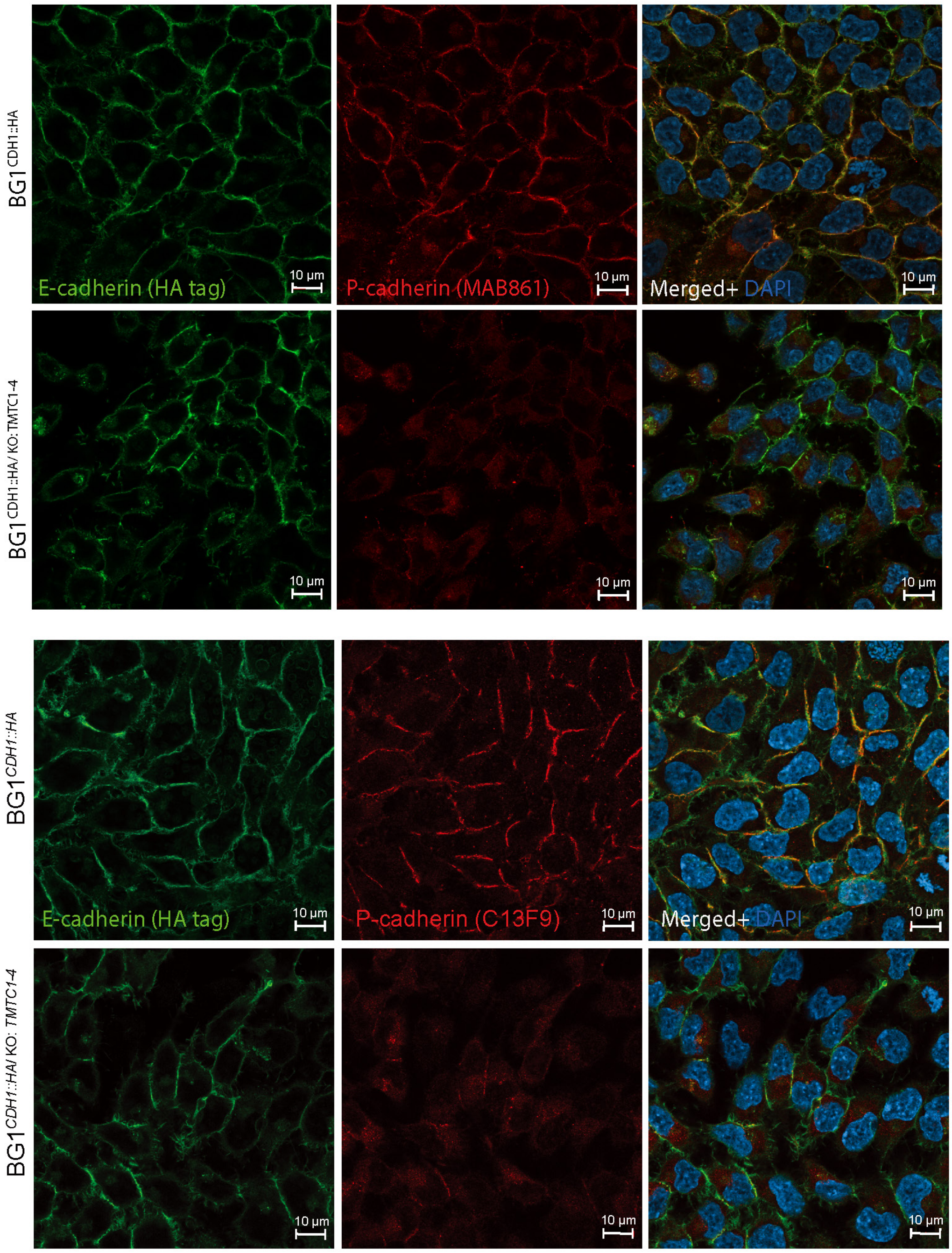

Supplemental Figure 12. Comparison of CDH1 and CDH3 localization in WT and *O*-Man depletion cells. Cells were stained for CDH1 via its HA-tag (green; anti-HA antibody 3F10) and for CDH3 (red; anti-CDH3 antibody MAB861 or C13F9). Nuclei were counterstained with DAPI (blue). Scale bar = 10 µm.
